# RETRACTED: Garza-Morales et al. Targeting Melanoma Hypoxia with the Food-Grade Lactic Acid Bacterium *Lactococcus lactis*. *Cancers* 2020, *12*, 438

**DOI:** 10.3390/cancers18111729

**Published:** 2026-05-26

**Authors:** Rodolfo Garza-Morales, Beatriz E. Rendon, Mohammad Tariq Malik, Jeannete E. Garza-Cabrales, Anne Aucouturier, Luis G. Bermúdez-Humarán, Kelly M. McMasters, Lacey R. McNally, Jorge G. Gomez-Gutierrez

**Affiliations:** 1 Department of Surgery, School of Medicine, University of Louisville, Louisville, KY 40202, USA; r0garz01@louisville.edu (R.G.-M.); jeannete.garza@gmail.com (J.E.G.-C.); kelly.mcmasters@louisville.edu (K.M.M.); 2 Molecular Targets Program, James Graham Brown Cancer Center, University of Louisville, Louisville, KY 40202, USA; beatriz.rendon@louisville.edu; 3 Department of Microbiology, School of Medicine, University of Louisville, Louisville, KY 40202, USA; tariq.malik@louisville.edu; 4 INRAE, AgroParisTech, Micalis Institute, Université Paris-Saclay, 78350 Jouy-en-Josas, France; anne.aucouturier@inra.fr (A.A.); luis.bermudez@inra.fr (L.G.B.-H.); 5 Department of Bioengineering, Stephenson Cancer Center, University of Oklahoma, Norman, OK 73019, USA; lacey_mcnally@ou.edu

The journal retracts the article titled, “Targeting Melanoma Hypoxia with the Food-Grade Lactic Acid Bacterium Lactococcus Lactis” [1], cited above.

Following publication, concerns were brought to the attention of the Editorial Office regarding the integrity of a number of figures presented in this article [1].

In accordance with the journal’s standard procedures, the Editorial Office and Editorial Board conducted an investigation, with input from the Research Integrity Office at the University of Oklahoma, which identified indications of inappropriate image editing and duplication in Figure 6B, including the reuse of an image from an earlier publication [2] by the same authorship group. Although the authors cooperated with the investigation, the explanations and supporting materials provided were not deemed sufficient by the Editorial Board to resolve the identified concerns. As a result, the Editorial Board has lost confidence in the reliability of the findings and has decided to retract this publication [1], as per MDPI’s retraction policy.

This retraction was approved by the Editor-in-Chief of the journal *Cancers*.

The authors have been informed of this retraction and have indicated their agreement with this decision.
